# Responsible Ownership and Welfare Indicators for Dogs and Cats in Rural Lithuania: Self-Reported Practices and In-Person Assessment

**DOI:** 10.3390/ani16192988

**Published:** 2026-09-23

**Authors:** Eglė Marija Ramanauskaitė, Snieguolė Malakauskienė, Tautvilė Šukytė, Alvydas Malakauskas

**Affiliations:** 1 Department of Veterinary Pathobiology, Faculty of Veterinary Medicine, Veterinary Academy, Lithuanian University of Health Sciences, LT-47181 Kaunas, Lithuaniaalvydas.malakauskas@lsmu.lt (A.M.); 2 Miško Trobelė Animal Shelter, LT-18189 Švenčionys District Municipality, Lithuania; 3 Department of Anatomy and Physiology, Faculty of Veterinary Medicine, Veterinary Academy, Lithuanian University of Health Sciences, LT-47181 Kaunas, Lithuania

**Keywords:** companion animals, dogs, cats, responsible pet ownership, animal welfare, preventive veterinary care, companion animal population management, rural communities

## Abstract

Dogs and cats are increasingly regarded as companion animals and family members, but companionship requires more than affection: it also entails responsibility for their health, reproduction, housing and behavioural needs. Responsible ownership and welfare in rural Baltic and Eastern European settings remain poorly studied. The study included 383 Lithuanian owners (338 rural, 45 urban) and welfare assessments of 85 animals from 53 rural households using an adapted Animal Welfare Assessment Grid. Animal numbers per household differed significantly by residence (*p* = 0.0008), with 73.1% of rural respondents keeping two or more animals versus 46.7% of urban respondents. Microchipping of all household animals was less often reported by rural than urban respondents (55.5% vs. 86.7%; *p* = 0.0003), as was rabies vaccination within the previous three years (67.1% vs. 90.9%; *p* = 0.0047). Neutering of all animals was likewise less common in rural households, although not significantly (50.6% vs. 68.9%; *p* = 0.0633). Main welfare concerns were environmental enrichment, body condition, frequency of exposure to stressors, behaviour during procedures and the living environment; older animals had poorer scores (*p* = 0.0004). Rural households kept more animals, while several responsible ownership practices were less consistently implemented, potentially amplifying welfare and population-management consequences and supporting rural education and preventive veterinary initiatives.

## Simple Summary

Dogs and cats kept as companion animals are increasingly treated as family members, but affection alone does not guarantee responsible care for their health, reproduction, living conditions and behavioural needs. These responsibilities may be fulfilled differently across social and geographical settings, yet rural companion animal ownership and welfare in the Baltic states and Eastern Europe remain poorly studied. This study compared ownership and care practices in rural and urban households in Lithuania and assessed companion animals (dogs and cats) in rural homes. Questionnaires were completed by 383 owners, and 85 animals from 53 rural households were examined. Rural respondents more often kept two or more animals and were less likely to report that all household animals were microchipped and vaccinated against rabies. Neutering of all animals was also less common in rural households, although this difference was not statistically significant. The main welfare concerns involved limited opportunities for stimulation and natural behaviour, body condition, frequency of exposure to stressors, behaviour during handling and procedures, and the living environment. Older animals had poorer welfare scores. Rural households kept more animals, while several responsible ownership practices were followed less consistently, meaning that gaps in care may affect more animals. Owner education and preventive veterinary services could improve welfare and support population management in rural communities.

## 1. Introduction

Dogs and cats are increasingly regarded as companions and family members rather than animals kept solely for practical purposes [1,2]. This shift has raised expectations regarding the quality of care and welfare provided to them. However, describing an animal as a companion does not in itself ensure that all of its needs are met, and responsibility is understood and enacted variably among owners [3,4]. Responsible ownership should therefore be examined through specific care and management practices rather than inferred from ownership or affection alone.

In this study, responsible pet ownership refers to the owner’s practices and decisions throughout an animal’s life that are intended to meet its needs and fulfill responsibilities to the animal and the wider community. These include responsible acquisition, microchipping and registration, preventive healthcare, reproductive management, appropriate nutrition and housing, control of unsupervised roaming, and timely veterinary care [3,5,6]. These practices support individual animal health and welfare, owner accountability and companion animal population management [6,7]. Veterinary involvement additionally provides opportunities for early disease detection and professional guidance on nutrition, reproduction and behaviour [8,9]. Nevertheless, some owners do not seek veterinary care when an animal appears healthy or when no immediate need is perceived [10], resulting in a more reactive than preventive approach to care.

How owners fulfil these responsibilities can influence aspects of animal welfare that may be less immediately visible than illness or injury. Contemporary animal-welfare approaches recognise that welfare is shaped by nutrition, physical health, the living environment, opportunities to perform normal behaviour and the animal’s resulting mental state [11]. Owners may underestimate body condition in both dogs and cats [12], while studies across companion animal species have identified variation in owners’ understanding of behavioural and environmental needs and recognition of stress-related signs [8,13,14]. Thus, animals may receive basic care while still experiencing unmet nutritional, behavioural, environmental or psychological welfare needs.

The extent to which these responsibilities are implemented may vary across social and geographical settings. Rural communities are particularly relevant in this context because companion animal ownership patterns, husbandry practices and access to preventive services may differ from those in urban areas. In Chile, rural households were more likely to keep companion animals and had more animals per person, while neutering and microchipping rates were lower than in urban areas [6]. In four underserved communities in the United States, rural communities had higher overall pet-keeping and dog-ownership rates than urban communities [15]. In Great Britain, estimated dog and cat densities were highest in major metropolitan areas, but the number of pets per household was generally highest in rural areas [16]. A national household survey similarly found that dogs were more likely to be owned by rural households and cats by semi-urban or rural households [17]. Research in Denmark has also shown that microchipping cats remained less prevalent in rural areas than in more densely populated areas [18]. Where more animals are kept per household, inconsistent microchipping, preventive care and reproductive control may affect a greater number of animals. However, rural residence should not itself be interpreted as evidence of poorer care. Rural–urban differences are likely to reflect a combination of animal numbers, husbandry traditions, socioeconomic circumstances, service availability and owners’ understanding of animal needs rather than place of residence alone.

In Lithuania, companion animal welfare and ownership are governed by the Animal Welfare and Protection Law adopted in 1997 [19], which sets general welfare requirements, prohibits neglect and cruelty, and since its 2021 amendment mandates the microchipping and registration of all dogs and cats. Mandatory disease control and anti-rabies vaccination is regulated under the Veterinary Law adopted in 1991 [20], the associated Government resolutions on requirements for rabies vaccination adopted in 2007 [21], and disease control obligations [22]. In Lithuania, approximately one-third of the population lives in rural areas [23]. Although the total dog and cat populations are unknown because a nationwide census has not been fully implemented, the national Companion Animal Register recorded 345,085 dogs and 188,971 cats as of 1 January 2026, with approximately 65% of registered companion animals located outside the municipalities of the three largest cities [24]. Taking into account the lower microchipping rates that have been reported in rural areas by other studies [6,18], it is possible that the number of companion animals living in rural areas may be considerably higher than official records suggest, making this population particularly important for animal-welfare and population-management research.

Previous Lithuanian studies have examined factors influencing the choice of veterinary services and selected aspects of service provision, including veterinary telemedicine [25,26]. However, little is known about rural–urban differences in companion animal numbers, acquisition, microchipping, preventive care, reproductive control, husbandry practices and veterinary-care use. A bibliometric analysis of the international dog and cat welfare literature identified the United Kingdom, Italy, Spain, Germany and Austria as its principal European contributors, suggesting that Baltic and Eastern European settings remain underrepresented [1]. To our knowledge, no previous Lithuanian study has combined a rural–urban comparison of owner practices with a direct, structured welfare assessment of dogs and cats living in rural households.

To address these gaps, the present study combined questionnaire data on owners, households and owned dogs and cats—hereafter collectively referred to as companion animals—with direct animal welfare assessment. The study aimed to compare companion animal numbers, selected ownership and care practices, husbandry practices and veterinary-care use between rural and urban households in Lithuania, to describe companion animal acquisition routes in the overall sample, and to identify the principal welfare concerns among dogs and cats assessed in rural households using an adapted Animal Welfare Assessment Grid (AWAG).

We hypothesised that rural respondents would report keeping more companion animals and less consistent implementation of selected responsible ownership practices than urban respondents, and that overall welfare scores would vary according to owner sociodemographic characteristics.

## 2. Materials and Methods

### 2.1. Study Design and Setting

The study was conducted in Lithuania between September 2025 and January 2026. It consisted of an anonymous online questionnaire and an in-person owner questionnaire combined with direct animal welfare assessment. A convenience sampling strategy was used.

Eligible participants were adults who owned at least one dog or cat. The study focused primarily on rural communities. The online questionnaire also included urban owners who accessed the openly distributed survey, whereas the in-person component and all direct animal welfare assessments were conducted exclusively among rural residents. Rural areas were defined according to the Organisation for Economic Co-operation and Development (OECD) criterion as localities with a population density below 150 inhabitants/km^2^ [27]. Respondents were asked to provide the name of their place of residence, which was classified as rural or urban using official population-density data.

### 2.2. Ethical Approval and Informed Consent

The study, including the questionnaire and the non-invasive assessment of privately owned dogs and cats, was conducted with the approval of the Bioethics Centre of the Lithuanian University of Health Sciences (approval no. 2025-BEC2-0990, 26 September 2025). Participation was voluntary, and all participants were informed about the purpose of the study, the intended use of the results in scientific publications, their right to withdraw, and the protection of their data.

No directly identifying personal information or exact residential addresses were collected. Before accessing and completing the online questionnaire, all respondents were required to read information about the study objectives and the processing of personal data. Proceeding to and completing the questionnaire was considered confirmation of informed consent.

Before the in-person questionnaire and animal assessment, participants also agreed to take part and permitted the non-invasive examination of their animals. The assessments involved external observation and examination only and did not include painful or invasive procedures. All information relating to participants and animals was anonymised before analysis.

### 2.3. Questionnaire Development and Pilot Testing

A study-specific owner questionnaire was developed in Lithuanian based on the study objectives and relevant literature, under the guidance of researchers with expertise in veterinary epidemiology, animal welfare and companion animal behaviour. The draft questionnaire underwent pilot testing with a small sample of dog and cat owners before the main data collection to assess the clarity of the questions and the suitability of the response options and was revised accordingly until the final version was produced. The same core questionnaire was used for the online and in-person surveys, with additional questions and the animal welfare assessment included during the household visits. The English version provided as Supplementary Materials S1 and S2 was prepared for publication and was not used for data collection.

### 2.4. Online Questionnaire

The anonymous online questionnaire was created using Google Forms and distributed through local Facebook groups between November 2025 and January 2026. Because the invitation was circulated through open social-media posts, the number of individuals who viewed or received the invitation could not be determined and a formal response rate could not be calculated. The questionnaire required approximately 10–15 min to complete.

The questionnaire contained closed, multiple-response and open-ended questions organised into four principal sections. The first section collected owner demographic information, including age, gender, education, employment status and place of residence. The second collected information about the animals kept in the household, including the numbers of dogs and cats and the routes through which animals had been acquired. The third addressed responsible ownership and preventive-care practices, including microchipping, rabies vaccination, parasite prevention, neutering, breeding, feeding and housing. The fourth addressed veterinary-care use, responses to illness or injury, reasons for veterinary visits, frequency of attendance, perceived barriers to care and sources of information regarding animal health, nutrition, care and welfare.

The English version of the questionnaire is provided as Supplementary Material S1.

### 2.5. In-Person Owner Survey

The in-person component used an assisted questionnaire. The researcher entered participants’ answers into Google Forms containing the same core questions as the online survey, supplemented with questions and observations required for the animal welfare assessment.

Potential participants were approached during three companion-animal microchipping and neutering campaigns and subsequently in individual cases in which owners requested veterinary assistance. All eligible adult dog or cat owners encountered through these activities were invited to participate, irrespective of owner or animal characteristics, and no additional selection parameters were applied. Participation was entirely voluntary, and the decision to participate, decline participation, or withdraw had no effect on access to or receipt of any services or assistance provided through these initiatives or by the shelter. Only adults who consented to both the questionnaire and the examination of at least one animal were included.

Each participating owner was assigned an anonymised identification number. Individual animal identification numbers were used to link the owner questionnaire with the relevant welfare assessment while maintaining anonymity. The combined owner interview and animal assessment generally required 15–20 min.

The English version of the questionnaire is provided as Supplementary Material S2.

### 2.6. Animal Welfare Assessment

Animal welfare was assessed using an adapted version of the Animal Welfare Assessment Grid (AWAG), which was previously preliminarily validated for dogs [28]. Within this tool, individual factors within the physical, psychological, environmental and procedural welfare parameters are scored from 1, representing the most favourable welfare state, to 10, representing the least favourable welfare state. Mean factor scores are calculated for each parameter and used to derive a polygon-based cumulative welfare assessment score.

For the present study, the method was adapted for a one-time assessment of both dogs and cats at a single time point. The underlying 1–10 scoring structure and severity thresholds were retained for both species, while species-specific behavioural expressions and needs were used when interpreting the descriptors. Written modifications included feline expressions, such as hissing and scratching, alongside canine growling and biting in the aggression indicators. During the structured interview, the assessor also explained species-appropriate manifestations of stress and species-specific environmental and behavioural needs, including hiding, scratching, exploration, hunting-related behaviour and enrichment in cats. Depending on the indicator, the score was either selected by the owner following this explanation or assigned by the assessor based on direct observation and owner-provided information. These species-specific interpretations were grounded in the assessor’s formal veterinary education and extensive hands-on experience in evaluating and managing the behavioural and welfare needs of dogs and cats. The adapted assessment form used in the study is provided as Supplementary Material S3.

All assessments were performed by the same veterinary medicine student using the predefined descriptors in the adapted assessment form. Thirteen welfare indicators were evaluated: mobility; body condition; clinical assessment; eating and drinking; aggression towards the caregiver; aggression towards unfamiliar people; fears and anxieties frequency; reaction to stressors; choice, control and predictability; enrichment; social interactions; behaviour during assessment; and handling during assessment.

Two factors included in the original AWAG—change in daily routine and procedure pain—were excluded because they were intended to evaluate the effects of veterinary or management procedures and were not applicable to the non-invasive, single-assessment design of the present study.

Each of the 13 indicators was assigned a numerical score using the corresponding verbal descriptor in the adapted assessment form. For the present analysis, a study-specific overall welfare score was calculated as the arithmetic mean of the 13 indicator scores. This arithmetic mean was used as a study-specific descriptive summary and did not represent the polygon-based cumulative score used in the original AWAG. Lower scores indicated more favourable welfare.

The direct welfare-assessment component was intentionally restricted to rural households because it was designed to identify welfare priorities in the study’s target rural population and was conducted through rural community initiatives; it was not intended to provide a rural–urban comparison of animal-level welfare.

### 2.7. Variable Definition and Data Processing

Responses from the online and in-person questionnaires were exported and combined into a single analytical database. Questions that were identical in both questionnaires were harmonised and analysed together.

Owner education was grouped as higher education, comprising university and college higher education, or no higher education, comprising primary, lower-secondary, secondary and vocational education. Employment status was grouped as employed or unemployed.

The total number of animals in each household was calculated by combining the reported numbers of dogs and cats and was grouped as one, two to five, six to ten, or eleven or more animals. The online questionnaire recorded the numbers of dogs and cats using predefined categories (0, 1, 2–5, 6–10, or ≥11), whereas the in-person questionnaire recorded exact counts. For the combined analysis, the exact in-person counts were recoded into the corresponding categories. After harmonisation, household animal numbers were analysed as one, two to five, six to ten, or eleven or more animals.

Household-level microchipping, rabies vaccination and neutering responses were harmonised into three categories: none of the animals, some of the animals, or all animals in the household. Rabies vaccination referred to vaccination within the previous three years.

For questions allowing multiple selections, each option was converted into a separate binary variable, coded as 0 when not selected and 1 when selected. Consequently, response categories for acquisition routes, feeding, housing, veterinary-care behaviour, barriers and information sources were not mutually exclusive. Open-text responses and responses entered under “other” were reviewed and, where appropriate, assigned to existing categories. Responses that could not be assigned to a predefined category were grouped according to their content by identifying recurrent themes.

Animal-welfare responses recorded using verbal descriptors were converted into numerical scores according to the adapted AWAG assessment form. Owner questionnaire data and animal-level welfare data were linked through anonymised identification numbers.

### 2.8. Statistical Analysis

Statistical analyses were performed using Microsoft Excel (Microsoft Corporation, Redmond, WA, USA) and JMP^®^ 18 (JMP Statistical Discovery LLC, Cary, NC, USA). Frequencies and percentages were calculated for categorical variables, while means, medians and ranges were calculated for continuous welfare scores. Pearson’s chi-square test was used to assess associations between categorical variables, including rural–urban differences and associations between owner demographic characteristics and household-level ownership-practice outcomes. To account for the non-independence of welfare scores from animals within the same household, welfare score comparisons were performed using linear mixed models (restricted maximum likelihood, REML) with household as a random intercept. Fixed effects were animal age (categorical: <1, 1–4, 5–9 and ≥10 years; one model) and each owner demographic characteristic (education, gender, age and employment status; one model each). Statistical significance was set at *p* < 0.05.

### 2.9. Use of Generative Artificial Intelligence

During the preparation of this manuscript, the authors used ChatGPT (OpenAI; GPT-5.5 and GPT-5.6 Thinking; https://chatgpt.com/; accessed on 30 July 2026) and Perplexity AI (Perplexity; https://www.perplexity.ai/; accessed on 30 July 2026) to assist with manuscript organisation and coherence across sections, linguistic refinement, preliminary identification of potentially relevant literature, and review of compliance with journal formatting and publication requirements. All generated outputs were critically reviewed, verified and edited by the authors, who take full responsibility for the final content of the manuscript.

## 3. Results

### 3.1. Study Population

A total of 383 cat and dog owners participated in the study, including 330 respondents who completed the online questionnaire and 53 who participated in the in-person survey. For the in-person component, 57 eligible owners were invited to participate; 53 (93.0%) agreed and completed the questionnaire, while four declined to complete it and were therefore not included. Overall, 338 (88.3%) respondents resided in rural areas and 45 (11.7%) in urban areas. Women represented 89.8% of respondents, while 65.6% had completed higher education. During the in-person survey, welfare assessments were performed on 85 companion animals, including 34 dogs and 51 cats. The demographic characteristics of the study population are presented in Table 1.

### 3.2. Pet Ownership Characteristics

Overall, 48.6% of respondents owned two to five companion animals, 30.0% owned a single animal, 15.7% owned six to ten animals, and 5.7% owned eleven or more animals. The distribution of companion animal numbers differed significantly between rural and urban households (χ^2^ = 16.675, df = 3, *p* = 0.0008; Figure 1). Nearly three-quarters of rural respondents (73.1%) owned two or more companion animals, compared with 46.7% of urban respondents. Conversely, more than half of urban households (53.3%) kept only one companion animal, whereas this was the case for only 26.9% of rural households.

Respondents aged 45 years and older also tended to own larger numbers of companion animals than younger respondents (χ^2^ = 9.977, df = 3, *p* = 0.0188). Employment status showed a similar tendency, with unemployed respondents more frequently reporting larger numbers of companion animals than employed respondents, although this association did not reach statistical significance (χ^2^ = 7.754, df = 3, *p* = 0.0514).

Companion animals were acquired predominantly through informal sources. Overall, 69.3% of acquisition-source responses represented informal routes, including animals taken in as strays, gifted by acquaintances or family, obtained through advertisements, born to already-owned animals, purchased from unregistered breeders, or acquired from private individuals. In comparison, shelter adoptions accounted for 15.3% of responses, purchases from registered breeders for 14.1%, and other sources for 1.4%. The distribution of acquisition sources is presented in Figure 2.

### 3.3. Microchipping, Vaccination and Neutering Practices

Microchipping, rabies vaccination and neutering practices were compared between rural and urban households (Figure 3). Complete microchipping was reported by 55.5% of rural respondents and 86.7% of urban respondents, while no pets were microchipped in 32.8% and 8.9% of households, respectively (χ^2^ = 15.987, df = 2, *p* = 0.0003). Complete rabies vaccination within the previous three years was reported by 67.1% of rural and 90.9% of urban respondents; no pets were vaccinated in 23.3% and 4.5% of households, respectively (χ^2^ = 10.742, df = 2, *p* = 0.0047). Complete neutering was reported by 50.6% of rural and 68.9% of urban respondents, whereas no pets were neutered in 31.7% and 17.8% of households, respectively; this difference did not reach statistical significance (χ^2^ = 5.518, df = 2, *p* = 0.0633). Routine parasite prevention was commonly reported in both groups, with no significant rural–urban difference (χ^2^ = 0.363, df = 1, *p* = 0.5471).

Higher education was associated with complete microchipping (67.6% vs. 43.1%; χ^2^ = 23.179, df = 2, *p* < 0.0001), rabies vaccination (78.2% vs. 53.5%; χ^2^ = 26.949, df = 2, *p* < 0.0001) and neutering (58.2% vs. 42.4%; χ^2^ = 16.617, df = 2, *p* = 0.0002). Employment was also associated with complete microchipping (62.2% vs. 47.4%; χ^2^ = 6.034, df = 2, *p* = 0.0489) and rabies vaccination (74.1% vs. 52.7%; χ^2^ = 13.474, df = 2, *p* = 0.0012). Rabies vaccination coverage additionally differed according to age (χ^2^ = 20.444, df = 10, *p* = 0.0253); among respondents aged 65 years and older, 48.1% reported that all pets were vaccinated, while the same proportion reported that none were vaccinated.

Commonly reported reasons for incomplete microchipping included a perceived lack of need, financial or logistical difficulties, the animal’s age, concerns about causing stress, difficulty catching the animal, limited knowledge and unwillingness to microchip. Reasons for incomplete rabies vaccination included a perceived lack of need because of the animal’s lifestyle or age, financial and logistical difficulties, problems catching or transporting the animal, limited knowledge, distrust of vaccines and postponement. Reasons for not neutering included a perceived lack of need, the animal’s age, breeding intentions, financial or logistical difficulties and concerns about possible health or behavioural effects.

Overall, 49.9% of respondents reported that they did not perceive a need to visit a veterinarian or to do so more frequently.

### 3.4. Husbandry Practices

Housing practices varied descriptively between residential settings. Because respondents could select more than one option, the reported categories were not mutually exclusive. Keeping animals indoors accounted for 93.6% of urban and 63.7% of rural housing responses. Unrestricted outdoor access was reported more frequently in rural than urban settings (20.6% vs. 4.3%), as was housing in outdoor kennels (11.2% vs. 2.1%). Tethering accounted for 4.5% of rural housing responses and was not reported by urban respondents.

Feeding categories were also non-mutually exclusive. Premium or super-premium commercial diets were reported more frequently in urban than rural households (51.3% vs. 31.3%). Rural respondents more frequently reported supermarket pet food (19.5% vs. 10.3%), table scraps (9.9% vs. 3.8%), bones (9.4% vs. 6.4%) and porridge (6.3% vs. 5.1%). These rural–urban comparisons are descriptive, as inferential statistical tests were not performed for these multi-response variables.

### 3.5. Animal Welfare Assessment

Animal welfare was assessed in 85 animals from 53 rural households (30 households with 1 animal, 17 with 2, 4 with 3, 1 with 4, 1 with 5), including 34 dogs (40.0%) and 51 cats (60.0%), using the adapted Animal Welfare Assessment Grid. The mean overall welfare score was 2.46 ± 0.88 in dogs (*n* = 34) and 2.18 ± 0.74 in cats (*n* = 51). Within the dog group, the highest mean indicator scores were recorded for enrichment (5.79 ± 3.53), body condition (3.03 ± 2.75), and choice, control and predictability (2.77 ± 2.09). Within the cat group, the highest mean indicator scores were recorded for enrichment (3.29 ± 3.32), fears and anxieties frequency (3.04 ± 2.18), body condition (3.00 ± 1.82), and choice, control and predictability (3.00 ± 1.95). Higher scores indicate poorer welfare.

Overall, the mean overall welfare score was 2.29, the median was 2.23, and scores ranged from 1 to 4.38, with lower scores indicating better welfare. The highest mean indicator scores were recorded for enrichment, body condition, handling during assessment, fears and anxieties frequency, and choice, control and predictability. The lowest mean indicator scores were recorded for aggression towards the caregiver and unfamiliar people, social interactions, mobility, and eating and drinking (Figure 4).

After accounting for household clustering (linear mixed model, household as random intercept), animal age remained significantly associated with the overall welfare score (F (4, 72.7) = 5.92, *p* = 0.0004). Animals younger than one year had the most favourable scores (estimate −0.727 relative to the reference group; *p* = 0.0004), and animals aged 10 years and older had the least favourable scores (estimate +0.581; *p* = 0.003). Animals in the intermediate age groups (1–4 and 5–9 years) did not differ significantly from the reference category (*p* = 0.183 and *p* = 0.273, respectively). Group means were consistent with the unadjusted analysis: animals aged <1 year, 1.69; 1–4 years, 2.22; 5–9 years, 2.17; ≥10 years, 2.99 (Figure 5). The intraclass correlation coefficient (ICC) for household was 14.2% in the animal age model. In contrast, the overall welfare score did not differ significantly by any owner demographic characteristic (education: F (1, 55.5) = 0.45, *p* = 0.507; gender: F (1, 68.7) = 0.75, *p* = 0.390; age: F (1, 56.9) = 0.36, *p* = 0.552; employment: F (1, 55.2) = 0.44, *p* = 0.511), with household ICC ranging from 21.6% to 23.9% across these models.

## 4. Discussion

The present study found that rural households kept more companion animals and reported less consistent implementation of microchipping and rabies vaccination than urban households. Neutering also appeared less complete in rural households, but the residential difference was not statistically significant. By contrast, the hypothesis that overall welfare scores would vary according to owner demographic characteristics was not supported. Microchipping, vaccination and reproductive control remain important elements of responsible ownership because they contribute to animal welfare, owner accountability and companion animal population management [3,7].

The tendency for rural households to keep more animals is consistent with studies from Chile, the United States and Ireland, where pet ownership has likewise been reported to be more common in rural than urban communities [6,15,29]. In Lithuania, only 31.6% of the population lives in rural areas, while approximately 64% of registered pets are located outside the three largest cities [23,24]. Given the lower microchipping rates observed in rural households, official records may substantially underestimate the true number of animals in these areas. Comparable patterns may also occur elsewhere in the Baltic and Eastern European regions with similar rural contexts, although further research is needed. This geographical distribution should be considered when planning microchipping, vaccination and reproductive control programmes, as well as broader responsible ownership and welfare interventions.

Microchipping was one of the practices showing the clearest rural–urban differences in the present study. Rural respondents were less likely than urban respondents to report that all companion animals in their household were microchipped. In Lithuania, dogs, cats and ferrets born on or after 1 May 2021 must be microchipped within four months of birth and before transfer to another owner or keeper, whereas animals born before that date had to be microchipped by 1 May 2022 [30]. Only animals registered in the Companion Animal Register may be transferred to another owner or keeper [30]. Microchipping and accurate registration support proof of ownership and legal accountability, facilitate the return of lost animals and provide traceability when animals are transferred [7,30,31,32]. Lower uptake may therefore reduce the likelihood that roaming or lost animals are returned to their owners and make it more difficult to identify the previous keeper when an animal is lost, abandoned or transferred informally. Similar rural–urban differences have been reported in Chile for dogs and cats and in Denmark for cats [6,18]. The associations with education and employment observed in the present study indicate that residence was not the only factor associated with microchipping. Possible explanations include differences in awareness, financial circumstances and access to registration services, but these mechanisms were not directly measured. Evidence from other compulsory microchipping systems also indicates that limited knowledge, confusion about registration databases and low engagement may reduce compliance even when a legal requirement exists [33].

Rabies vaccination also differed between residential settings, with rural respondents less likely to report that all animals in the household had been vaccinated within the previous three years. Regular rabies vaccination of dogs, cats and ferrets is mandatory in Lithuania. Comparable requirements also exist in Estonia and neighbouring Poland, although the species covered and revaccination intervals differ [34,35,36]. The lower reported coverage therefore indicates incomplete uptake of legally required vaccination among some respondents. Vaccination was also associated with education and employment, suggesting that residence was not the only relevant factor; however, the possible roles of awareness, financial circumstances and access to veterinary services were not directly assessed. Maintaining vaccination coverage remains important because infected animals may be imported from endemic areas, as illustrated by a 2026 case involving a dog brought from Russia to Germany [37]. Rabies vaccination therefore represents both an individual animal-health responsibility and a population-level One Health measure. Improving uptake may require accessible services together with targeted communication about owners’ legal and public-health responsibilities [7].

Neutering showed only a non-significant rural–urban tendency. Rural respondents were less likely to report that all animals in the household had been neutered and more likely to report that none had been neutered, but the present study did not demonstrate a statistically significant residential difference. Higher neutering rates in urban areas than in rural areas have previously been reported in Chile [6]. In the present study, education was more clearly associated with neutering than place of residence. Previous research has identified veterinary advice, perceived owner responsibility and expected health or welfare outcomes as factors influencing decisions about neutering [38]. Targeted, evidence-based communication may therefore help owners make informed reproductive-management decisions, although the reasons underlying the observed association with education were not directly assessed in the present study.

It is worth noting that urban respondents more frequently kept a single animal than rural respondents (53.3% vs. 26.9%); therefore, the “all animals” criterion was easier to meet in urban households because fewer animals had to be microchipped, vaccinated or neutered. The observed rural–urban differences in these practices may thus partly reflect differences in the number of animals kept per household rather than residence alone. Furthermore, because ownership and care practices were reported at the household rather than species level, the contribution of household species composition to the observed rural–urban differences could not be determined.

Companion animals were acquired predominantly through informal routes. Overall, 69.3% of acquisition-source responses represented animals taken in as strays, gifted by acquaintances or family, obtained through advertisements, born to already-owned animals, purchased from unregistered breeders or acquired from other private individuals. Similar reliance on gifts, community networks, private transfers and strays has been reported in other countries [39,40,41,42]. Some of these routes may involve unplanned litters or transfers occurring outside formal identification and registration procedures. However, the present study did not link individual acquisition sources with subsequent microchipping, registration or neutering, and therefore cannot establish that informally acquired animals received less consistent care. The predominance of informal acquisition should instead be interpreted as identifying a potentially important point for population-management interventions, including microchipping before transfer, accurate recording of ownership changes and prevention of unplanned litters [7,43].

Nearly half of respondents overall did not perceive a need to visit a veterinarian or to do so more frequently. This finding may be consistent with a more reactive approach to veterinary care, in which professional advice is sought mainly when owners perceive an immediate health problem. Regular veterinary care can provide opportunities not only for vaccination and early disease detection, but also for evidence-based guidance on nutrition, behaviour, environmental enrichment, stress and other aspects of physical and psychological welfare [8,9,10,11]. When veterinary care is not sought regularly, opportunities for owner education and early recognition of less visible welfare concerns may be reduced. Increasing service availability alone may therefore be insufficient unless owners are also informed about the veterinarian’s role in maintaining health and welfare before overt disease develops [8,44].

Husbandry practices also differed between residential settings and revealed several concerning patterns in rural areas. Animals in rural households were less often kept exclusively indoors and were more commonly allowed unrestricted outdoor access, housed in outdoor kennels or tethered. Tethering is of particular welfare concern because prolonged restraint may restrict exercise, behavioural choice and social contact while increasing the risk of frustration, injury and exposure to adverse environmental conditions [45]. These concerns are increasingly reflected in European legislation: Austria prohibits keeping dogs on a chain or otherwise tethered, apart from short-term tethering in front of a shop, while the EU regulation adopted in 2026 will, once applicable, prohibit operators from tethering dogs or cats for more than one hour, except during medical treatment or participation in shows, exhibitions and competitions; limited exemptions may also apply to dogs intended for military, police or customs services [46,47]. Feeding practices also differed, with urban respondents more frequently reporting premium or super-premium commercial diets, whereas rural respondents more often reported supermarket pet food, table scraps, bones and porridge. Reliance on home-prepared food and table scraps has also been reported in other companion animal populations [48,49]. Appropriate nutrition is a core responsible ownership obligation, as owners must provide a complete and balanced diet suited to the animal’s species, life stage and health status. When table foods or unformulated home-prepared foods constitute a substantial part of the diet, they may dilute essential nutrients, provide excessive energy or expose animals to other nutritional risks [48,50,51]. These findings indicate that responsible ownership interventions in rural communities should address not only preventive healthcare but also everyday housing, freedom of movement, social opportunities and evidence-based feeding practices.

The direct welfare assessment identified comparatively higher mean indicator scores for enrichment, body condition, handling during assessment, fears and anxieties frequency, and choice, control and predictability. Because no validated cut-off exists for classifying the study-specific adapted overall score as favourable or unfavourable, these findings should be interpreted comparatively rather than as evidence that overall welfare was generally favourable. The results identify nutritional, behavioural and environmental aspects that may require attention, but the study did not assess owners’ recognition or understanding of these needs. The body-condition result is relevant to previous evidence that owners may underestimate excess body condition, while the environmental and behavioural findings are consistent with reported limitations in owners’ knowledge of feline behaviour, environmental requirements and stress-related signals [12,13,52]. Regular veterinary guidance is therefore important not only for disease prevention, but also for weight management, environmental enrichment and behavioural welfare [9,50,53]. Contrary to our hypothesis, within the rural welfare-assessment sample, the overall welfare score was not significantly associated with owner age, education, gender or employment status. Older animals received poorer overall welfare scores. Chronic disease, pain and age-related functional or behavioural changes may contribute to poorer welfare in senior animals; however, these factors were not directly assessed as explanatory variables in the present study, and their contribution to the observed association cannot be determined. Poorer welfare is not an inevitable consequence of aging, as early detection, preventive care and age-appropriate management can help preserve quality of life [54]. As the dog-specific AWAG was preliminarily validated for dogs, its adaptation for cats in the present study should be considered when interpreting the combined-species results [28].

Taken together, these findings show that the owner–animal relationship is expressed not only through the decision to keep an animal, but also through the everyday responsibilities assumed for its health, traceability, reproduction, housing, nutrition and behavioural welfare. In rural settings, the combination of larger household animal populations, lower compliance with microchipping and vaccination requirements, weaker reproductive control, informal transfers, limited engagement with preventive veterinary care and concerning husbandry practices may amplify both welfare and population-management consequences. This pattern indicates that responsible ownership represents an important practical dimension of the human–animal relationship and cannot be inferred from ownership alone. Effective responses should therefore combine legal accountability, accessible preventive services, traceable ownership transfer, reproductive control and practical education addressing both physical and psychological needs. Similar challenges may be relevant across other rural contexts, particularly where companion animals are numerous but formal ownership and preventive-care systems remain inconsistently applied.

### Study Limitations

Several limitations should be acknowledged. The convenience-sampling design does not permit causal conclusions and restricts the generalisability of the findings. The online questionnaire was distributed through open social-media posts, whereas in-person participants were recruited during microchipping and neutering campaigns and through requests for veterinary assistance. Both recruitment routes may have resulted in self-selection. The smaller urban comparison group reduced the precision of urban estimates and the statistical power to detect smaller rural–urban differences; therefore, non-significant comparisons should not be interpreted as evidence of equivalence. Rural–urban differences should be interpreted as comparisons within the study sample rather than as nationally representative estimates. In addition, the use of an online self-administered questionnaire and an assisted in-person questionnaire may have introduced survey-mode effects. Residence was classified using self-reported locality information or broad residential descriptions, and some misclassification may therefore have occurred.

Most ownership and husbandry practices were self-reported at household level and were not independently verified. Individual acquisition routes could not be linked to specific animals or their subsequent care, while the housing and feeding variables permitted multiple responses and were analysed descriptively. The direct welfare assessment included 85 animals from 53 rural households recruited through community initiatives and requests for assistance. This non-random subsample was intended to identify welfare priorities and cannot provide population-level prevalence estimates. Welfare was assessed at a single time point, so temporal variation could not be examined. Behavioural responses observed during the assessment, including defensive responses towards the unfamiliar assessor, may reflect the immediate assessment context rather than the animal’s usual behaviour or longer-term welfare state.

The AWAG was previously preliminarily validated for dogs and was adapted for cats in the present study. Although species-specific behavioural expressions and needs were considered during its administration, the adapted assessment did not undergo independent feline-specific expert review or formal validation. This limits interpretation of the combined-species findings and direct comparisons between dogs and cats. Moreover, the relatively small welfare-assessment sample, comprising 34 dogs and 51 cats, was not sufficient to support reliable species-specific comparisons. Future studies should use larger species-stratified samples to examine differences in the welfare needs and outcomes of dogs and cats.

## 5. Conclusions

This study highlights a mismatch between the scale of companion animal ownership and the consistent implementation of responsible ownership practices in rural Lithuania. The findings suggest that ownership alone does not ensure that animals’ health, behavioural and welfare needs are adequately met, particularly where larger numbers of animals are kept and ownership transfers remain informal.

Improving companion animal welfare in rural areas will require better microchipping and vaccination coverage, reproductive control and more regular preventive veterinary care. Owners also need practical guidance on nutrition, behaviour and animals’ psychological needs. Similar challenges may be relevant in other rural Baltic and Eastern European settings, but comparative research is needed to reveal how widely these patterns apply.

## Figures and Tables

**Figure 1:**
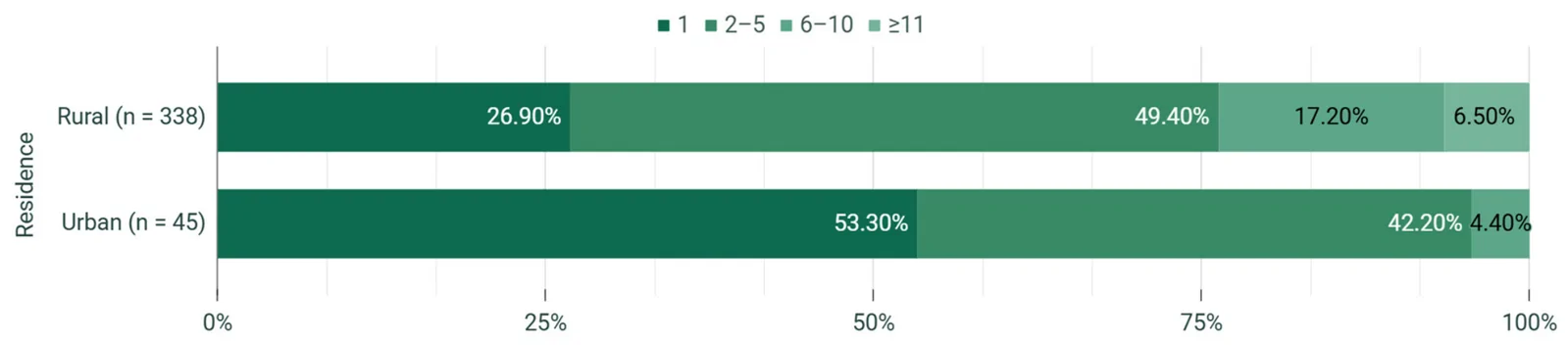
Distribution of the reported total number of companion animals per household by place of residence (rural vs. urban). Bars show within-group percentages (rural, *n* = 338; urban, *n* = 45). The distributions differed significantly between the groups (*p* = 0.0008).

**Figure 2:**
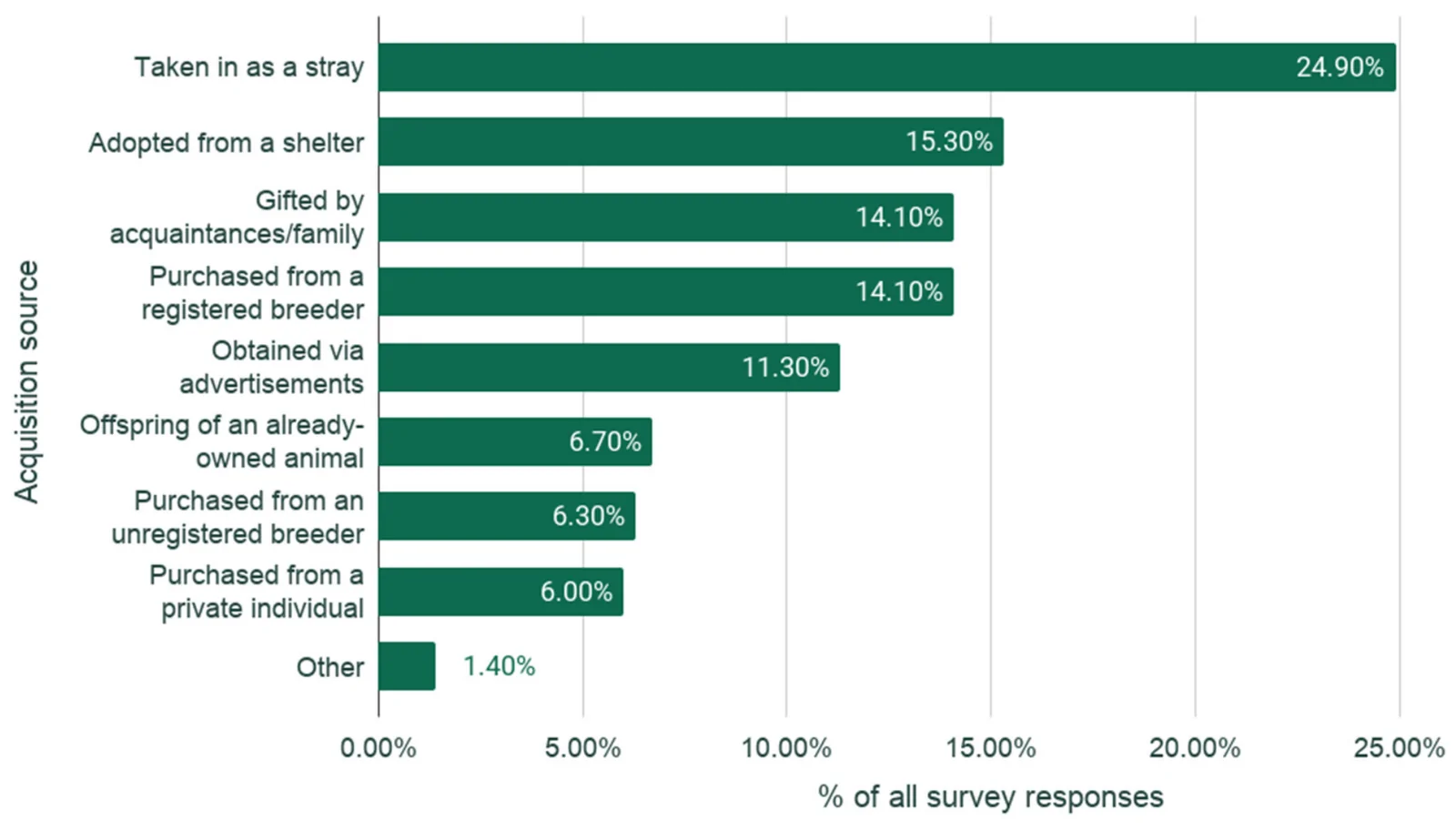
Distribution of reported companion animal acquisition sources. Percentages were calculated from all acquisition-source responses (*n* = 583) provided by 383 respondents; multiple responses were permitted.

**Figure 3:**
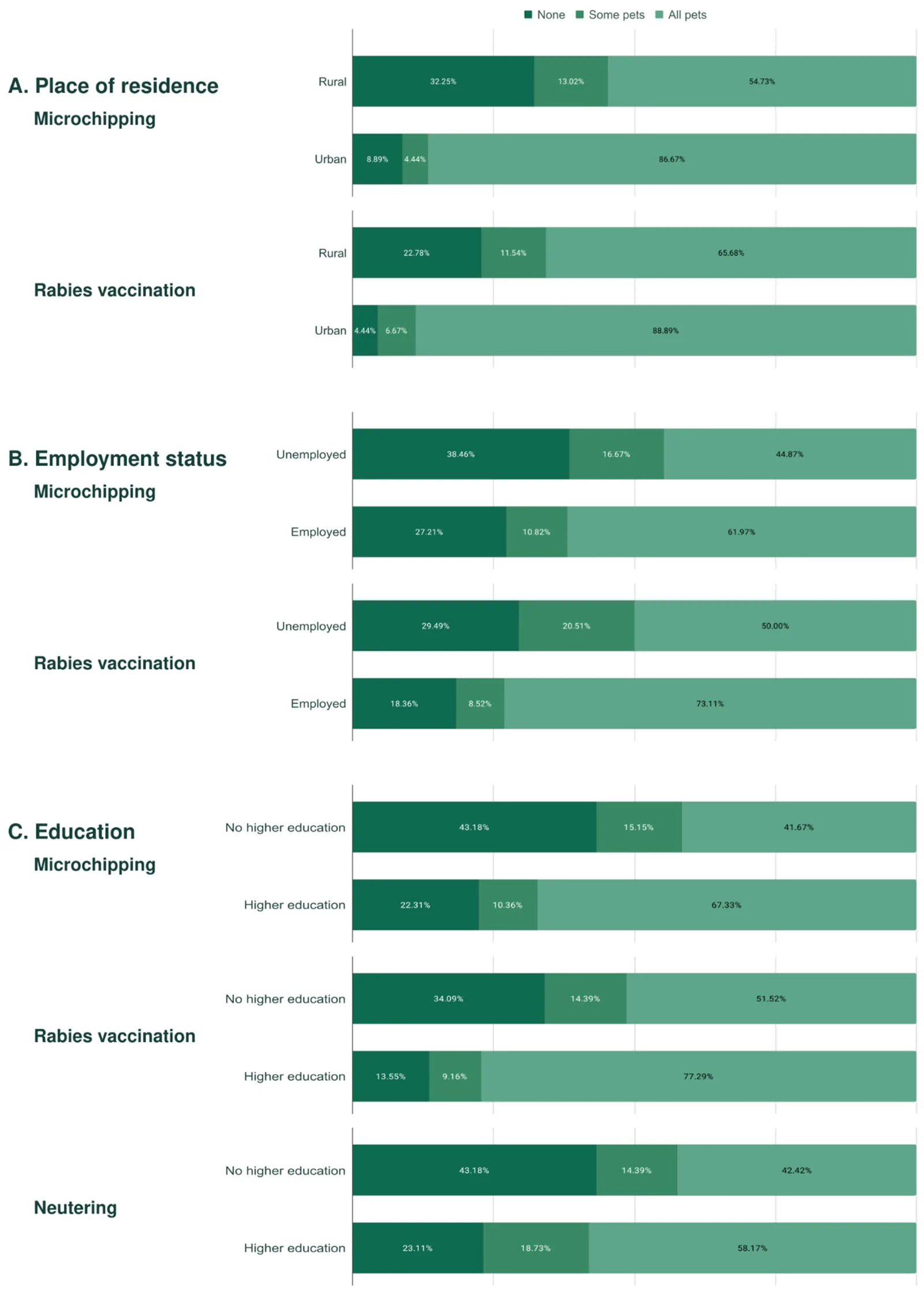
Distribution of selected preventive ownership practices according to (**A**) place of residence, (**B**) employment status, and (**C**) education. Bars show the percentage of respondents reporting that none, some, or all companion animals in their household were microchipped, vaccinated against rabies within the previous three years, or neutered. Pearson’s chi-squared tests: (**A**) microchipping, *p* = 0.0008; rabies vaccination, *p* = 0.0133; (**B**) microchipping, *p* = 0.0151; rabies vaccination, *p* = 0.0005; (**C**) microchipping, *p* < 0.0001; rabies vaccination, *p* < 0.0001; neutering, *p* = 0.0002.

**Figure 4:**
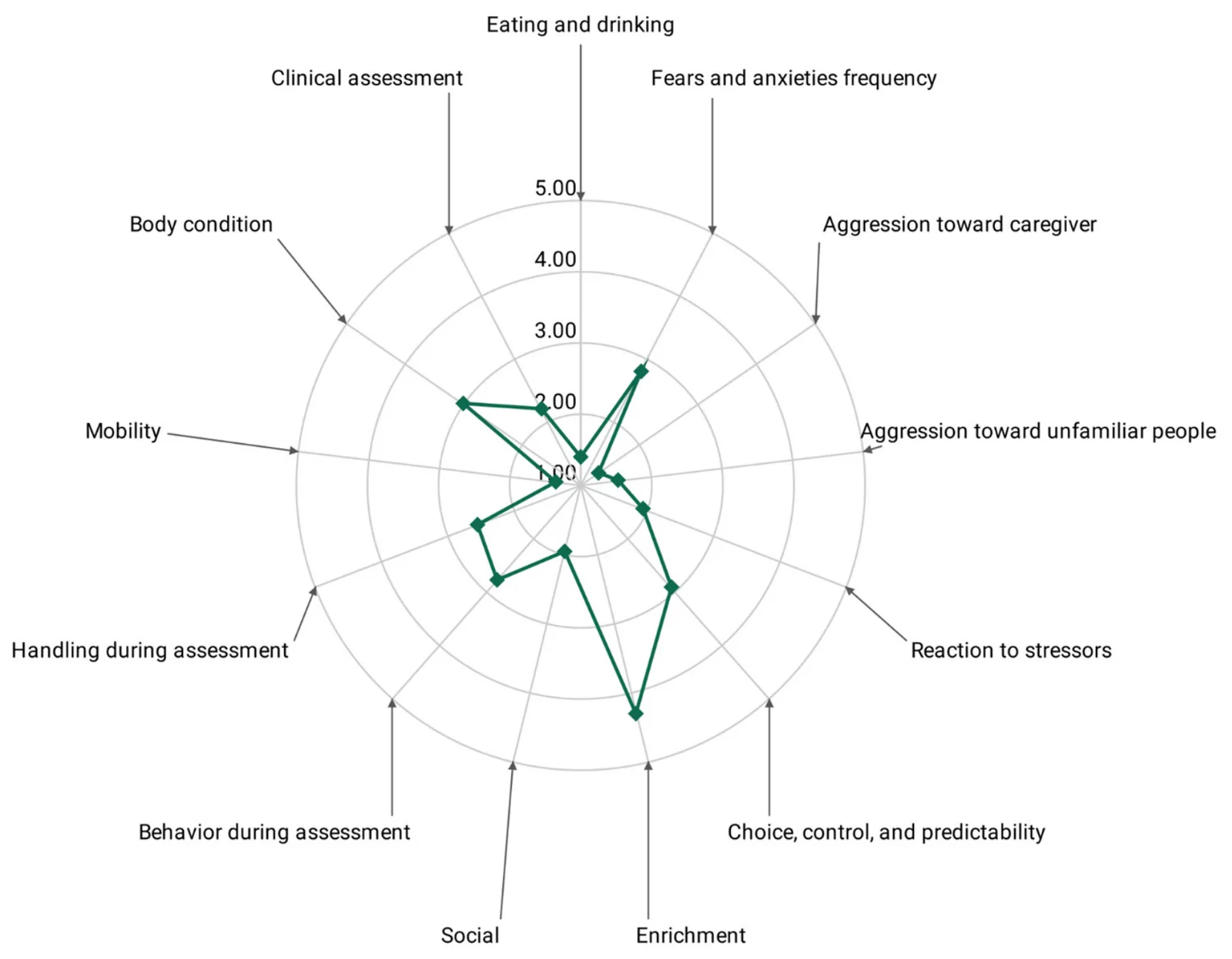
Mean scores for the 13 animal welfare indicators assessed using the Animal Welfare Assessment Grid (AWAG) among animals from rural households. Higher scores indicate poorer welfare.

**Figure 5:**
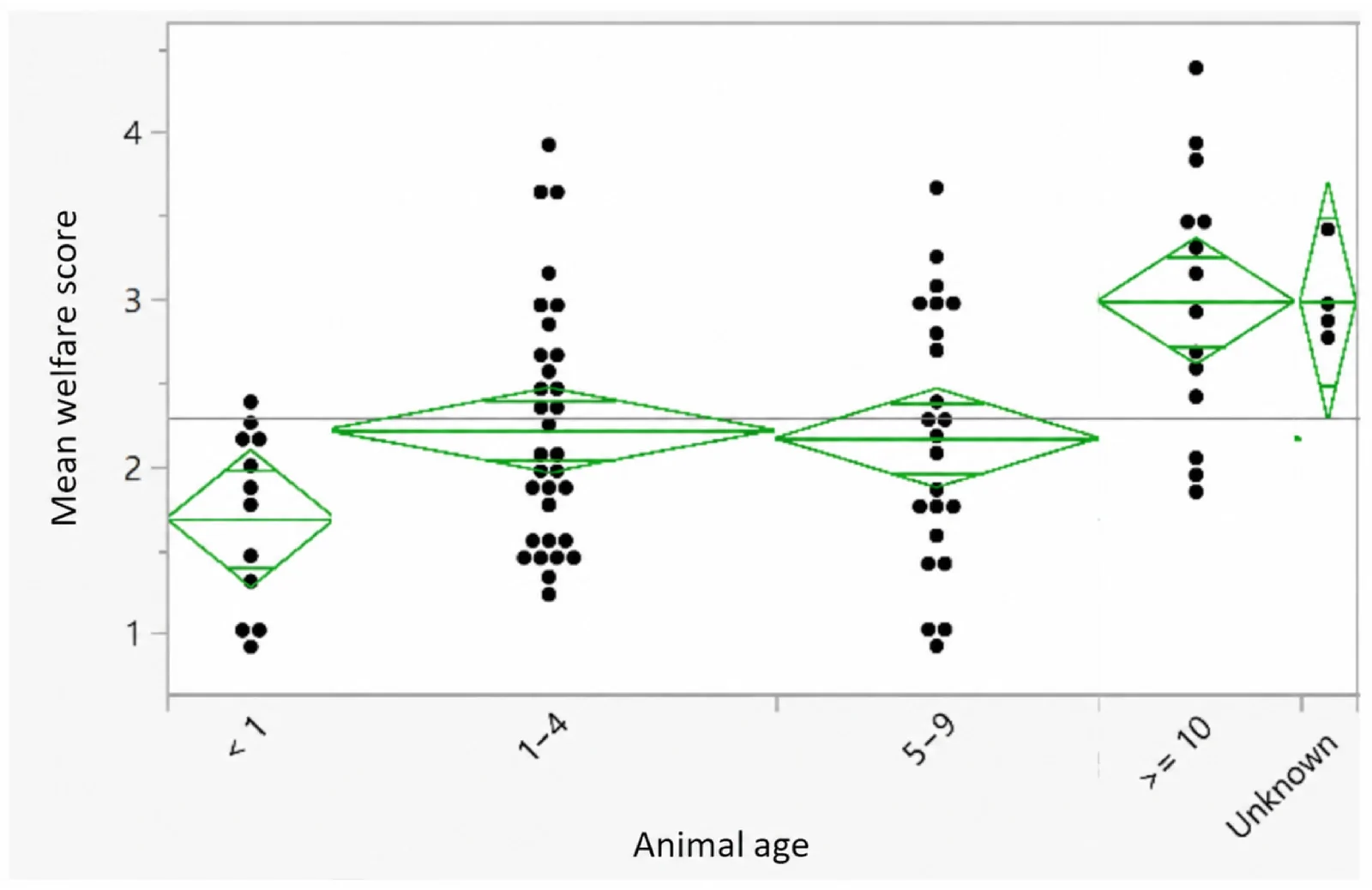
Distribution of mean welfare scores according to animal age group. Black dots represent individual animals, green diamonds indicate group means and their 95% confidence intervals, and the horizontal grey line represents the overall mean welfare score. Mean welfare scores differed significantly among age groups (*p* = 0.0004).

**Table 1:** Survey and sociodemographic characteristics of respondents (N = 383). Values are presented as number and percentage of respondents, *n* (%).

Characteristic		*n* (%)
**Survey type**	Online survey	330 (86.2)
	In-person survey	53 (13.8)
**Place of residence**	Rural	338 (88.3)
	Urban	45 (11.7)
**Gender**	Female	344 (89.8)
	Male	38 (9.9)
	Other	1 (0.3)
**Age group**	18–24	42 (11.0)
	25–34	64 (16.7)
	35–44	103 (26.9)
	45–54	96 (25.1)
	55–64	49 (12.8)
	≥65	29 (7.6)
**Education**	Higher education	251 (65.6)
	No higher education	132 (34.4)
**Employment status**	Employed	305 (79.6)
	Unemployed	78 (20.4)

## Data Availability

The anonymized data supporting the findings of this study are available from the corresponding author upon reasonable request. The data are not publicly available because they contain detailed locality- and household-level information that may increase the risk of participant re-identification.

## Supplementary Materials

The following supporting information can be downloaded at: https://www.mdpi.com/article/10.3390/ani16192988/s1, Supplementary Material S1. English Translation of the Online Questionnaire; Supplementary Material S2. English Translation of the In-Person Owner Questionnaire. Supplementary Material S3. Adapted Animal Welfare Assessment Form Based on the Animal Welfare Assessment Grid (AWAG).

## Abbreviations

The following abbreviations are used in this manuscript:

AWAG	Animal Welfare Assessment Grid
OECD	Organisation for Economic Co-operation and Development
