# Supplementary material for: Responsible Ownership and Welfare Indicators for Dogs and Cats in Rural Lithuania: Self-Reported Practices and In-Person Assessment
Source: Animals (Basel). 2026 Sep 23;16(19):2988. doi: 10.3390/ani16192988 (PMC13663609; doi:10.3390/ani16192988)
Supplement: Supplementary file 1 [file animals-16-02988-s001.zip › Supplementary Material S1.pdf]

Supplementary Material S1

English Translation of the Online Questionnaire

Participant Information

I am Eglė Marija Ramanauskaitė, a Veterinary Medicine student at the Lithuanian University of Health Sciences. I am conducting my Master's thesis entitled "Pet Owners' Access to Veterinary Services and Its Impact on Animal Welfare in Rural Areas of Lithuania".

You are invited to complete this anonymous questionnaire about the companion animals you keep and your experience of access to veterinary services. The survey data will help to clarify the factors influencing owners' decisions to seek or not to seek veterinary care and the potential barriers they encounter.

Participation in this study is entirely voluntary. You may decline to participate or withdraw your consent at any time. The survey is fully anonymous, and the data will be used only for scientific purposes—in the Master's thesis and in potential scientific publications.

The questionnaire takes approximately 10–15 minutes to complete.

The study was conducted with approval from the Bioethics Centre of the Lithuanian University of Health Sciences.

A. Owner Information

1. Owner age.

[Single response]

- 18–24
- 25–34
- 35–44
- 45–54
- 55–64
- 65 or older
- Other

2. Owner sex.

[Single response]

- Female
- Male
- Other

3. Owner education.

[Single response]

- Primary education
- Lower-secondary education
- Secondary education
- Vocational education
- Non-university higher education
- University higher education
- Other

4. Owner labour-market status.

[Multiple responses permitted]

- Employed

- Unemployed
- Reduced work capacity
- Retired
- Student
- Other

5. Place of residence (name of the village, town, or city; exact address not required).

[Open response]

____

B. Companion Animals Kept

6. Do you keep companion animals: cats and/or dogs?

[Single response]

- Yes
- No

C. Information about Companion Animals Kept

7. How many dogs do you keep?

[Single response]

- 0
- 1
- 2–5
- 6–10
- 11 or more

8. How many cats do you keep?

[Single response]

- 0
- 1
- 2–5
- 6–10
- 11 or more

9. Do you regularly care for animals other than your own? If so, which animals?

[Multiple responses permitted]

- I feed and care for free-roaming or feral cats
- I care for animals belonging to relatives or friends
- Sometimes
- I do not care for other animals
- Other

10. How did your companion animal(s) come into your care? Select all applicable answers for all animals you keep.

[Multiple responses permitted]

- Offspring of my own animal
- Given to me by acquaintances or family members
- Acquired from people through advertisements

- Took in an unowned animal from the street
- Adopted from an animal shelter
- Purchased from a registered breeder with documentation
- Purchased from a breeder without documentation
- Purchased from private individuals
- Other

11. Are your companion animal(s) microchipped?

[Single response]

- Yes
- No
- Some
- Do not know

12. If all or some companion animals are not microchipped, state why.

[Open response]

____

13. Were your companion animal(s) vaccinated against rabies within the past 3 years?

[Single response]

- Yes
- No
- Some
- Do not know

14. If all or some companion animals were not vaccinated against rabies, state why.

[Open response]

____

15. Do you regularly use antiparasitic products?

[Multiple responses permitted]

- We do not use them
- Yes, against worms (endoparasites)
- Yes, against ectoparasites (fleas and ticks)
- Do not know
- Other

16. If you do not use antiparasitic products, state why.

[Open response]

____

17. Are your companion animal(s) neutered?

[Single response]

- Yes
- No
- Some
- Do not know

18. If all or some companion animals are not neutered, state why.

[Open response]

____

19. Are your companion animal(s) used for breeding? Select all applicable answers for all animals you keep.

[Multiple responses permitted]

- Yes, there is a registered breeding establishment (kennel/cattery)
- Yes, but the breeding establishment is not registered
- The animal(s) are not used for breeding
- Accidental mating sometimes occurs
- There have been unplanned litters (females)
- Other

20. How many litters have your companion animal(s) had? Females only.

[Open response]

____

21. What happens to the offspring? Select all applicable answers.

[Multiple responses permitted]

- Kept by the owner
- Given to relatives or acquaintances
- Given away through advertisements
- Sold
- Surrendered to an animal shelter
- Other

22. What are your companion animal(s) fed? Select all applicable answers for all animals you keep.

[Multiple responses permitted]

- Commercial pet food from a grocery shop (e.g., Pedigree, Whiskas, etc.)
- Pet food from a pet shop (premium or super-premium class)
- Raw meat
- Bones
- Porridge
- Human food scraps
- Other

23. Where are your companion animal(s) kept? Select all applicable answers for all animals you keep.

[Multiple responses permitted]

- Indoors
- Outdoors, in an enclosure
- Outdoors, chained or tethered
- Outdoors, roaming freely
- Other

D. Animal Health and Access to Veterinary Services

24. What do you do if your companion animal(s) become ill or injured?

[Multiple responses permitted]

- Treat the animal myself (describe how in the comments)
- Give over-the-counter medicines (provide details in the comments, including whether human or veterinary medicines are used)
- Give prescription medicines or antibiotics obtained without a prescription (provide details in the comments, including whether human or veterinary medicines are used)
- Take the animal to a veterinarian
- Seek help from family members or acquaintances
- Seek help from animal-welfare organisations or shelters
- Do nothing
- Other

25. How often do you visit a veterinarian with your companion animal(s)?

[Single response]

- Never
- Once ever
- Once per year
- Twice or more per year
- Do not know
- Other

26. For what reasons do you visit a veterinarian with your companion animal(s)?

[Multiple responses permitted]

- Parasite prevention (worms, fleas, and ticks)
- Vaccination
- Neutering
- Illness
- Injury or trauma
- I do not visit a veterinarian
- Other

27. Why do you not visit, or only rarely visit, a veterinarian?

[Multiple responses permitted]

- The animal(s) have no need
- I do not perceive a need / in my opinion, a veterinarian is unnecessary
- Lack of money
- Lack of transport
- There is no veterinarian nearby
- The veterinarian's or clinic's opening hours are unsuitable
- I do not trust veterinarians
- Health or physical limitations
- Language barrier
- Do not know

- Other

28. Where do you obtain information about the care, nutrition, health, and welfare of your companion animal(s)?

[Multiple responses permitted]

- Veterinarian
- Veterinary pharmacy or pet-shop consultant
- Animal-welfare organisations or shelters
- Family, colleagues, or acquaintances
- Internet searches
- Social media
- Books
- Courses and training
- Other
