# Supplementary material for: Responsible Ownership and Welfare Indicators for Dogs and Cats in Rural Lithuania: Self-Reported Practices and In-Person Assessment
Source: Animals (Basel). 2026 Sep 23;16(19):2988. doi: 10.3390/ani16192988 (PMC13663609; doi:10.3390/ani16192988)
Supplement: Supplementary file 1 [file animals-16-02988-s001.zip › Supplementary Material S2.pdf]

Supplementary Material S2

English Translation of the In-Person Owner Questionnaire

Participant Information

I am Eglė Marija Ramanauskaitė, a Veterinary Medicine student at the Lithuanian University of Health Sciences. I am conducting my Master's thesis entitled "Pet Owners' Access to Veterinary Services and Its Impact on Animal Welfare in Rural Areas of Lithuania".

The aim of the study is to assess how access to veterinary services affects animal health and welfare in rural areas. During the study, pet owners are asked about their experiences and decisions concerning animal care, and companion-animal welfare is assessed using a standardised method. There is no direct harm or risk to you or your animal: only an external, painless examination of the animal is performed. The study was conducted with approval from the Bioethics Centre of the Lithuanian University of Health Sciences.

Participation in this study is entirely voluntary. You may decline to participate or withdraw your consent at any time. No personally identifying data are collected, and all information about you and your animal is anonymised. The collected data will be used only for scientific purposes—in the Master's thesis and in potential scientific publications.

The questionnaire and assessment take approximately 15–20 minutes.

If you have questions or wish to withdraw your consent, you may contact me at eglram1201@lsmu.lt.

Case ID: ______________________________ [completed by the researcher]

A. Owner Information

1. Owner age.

[Single response]

- 18–24
- 25–34
- 35–44
- 45–54
- 55–64
- 65 or older
- Other

2. Owner sex.

[Single response]

- Female
- Male
- Other

3. Owner education.

[Single response]

- Primary education
- Lower-secondary education
- Secondary education
- Vocational education

- Non-university higher education
- University higher education
- Other

4. Owner labour-market status.

[Multiple responses permitted]

- Employed
- Unemployed
- Reduced work capacity
- Retired
- Student
- Other

5. Place of residence (name).

[Open response]

____

B. Information about Companion Animals Kept

6. How many dogs do you keep?

[Open response]

____

7. How many cats do you keep?

[Open response]

____

8. Do you regularly care for animals other than your own? If so, which animals?

[Multiple responses permitted]

- I feed and care for free-roaming or feral cats
- I care for animals belonging to relatives or friends
- Other

C. Animal Health and Access to Veterinary Services

9. What do you do if your companion animal(s) become ill or injured?

[Multiple responses permitted]

- Treat the animal myself (describe how in the comments)
- Give over-the-counter medicines (provide details in the comments, including whether human or veterinary medicines are used)
- Give prescription medicines or antibiotics obtained without a prescription (provide details in the comments, including whether human or veterinary medicines are used)
- Take the animal to a veterinarian
- Seek help from family members or acquaintances
- Seek help from animal-welfare organisations or shelters
- Do nothing
- Other

10. How often do you visit a veterinarian with your companion animal(s)?

[Multiple responses permitted]

- Never
- Once ever
- Once per year
- Twice or more per year
- Do not know
- Other

11. For what reasons do you visit a veterinarian with your companion animal(s)?

[Multiple responses permitted]

- Parasite prevention (worms, fleas, and ticks)
- Vaccination
- Neutering
- Illness
- Injury or trauma
- Other

12. Why do you not visit, or only rarely visit, a veterinarian?

[Multiple responses permitted]

- I do not perceive a need
- Lack of money
- Lack of transport
- There is no veterinarian nearby
- The veterinarian's or clinic's opening hours are unsuitable
- I do not trust veterinarians
- Health or physical limitations
- Language barrier
- Do not know
- Other

13. Where do you obtain information about the appropriate care, nutrition, health, and welfare of your companion animal(s)?

[Multiple responses permitted]

- Veterinarian
- Veterinary pharmacy or pet-shop consultant
- Animal-welfare organisations or shelters
- Family, colleagues, or acquaintances
- Internet searches
- Social media
- Books
- Courses and training
- Other

D. Information about the Companion Animal (Complete for Each Animal)

14. Species.

[Single response]

- Dog
- Cat

15. Breed.

[Single response]

- Mixed-breed
- Other

16. Sex.

[Single response]

- Female
- Male

17. Age.

[Open response]

____

18. How long has the animal been in your care?

[Single response]

- Less than 1 year
- 1–3 years
- 4–5 years
- 5 years or more

19. How did the animal come into your care?

[Single response]

- Offspring of my own animal
- Given to me by acquaintances or family members
- Acquired from people through advertisements
- Took in an unowned animal from the street
- Adopted from an animal shelter
- Purchased from a registered breeder with documentation
- Purchased from a breeder without documentation
- Purchased from private individuals
- Other

20. Is the animal microchipped? The assessor checks for a microchip.

[Single response]

- Yes
- No
- Performed during the study

21. Are your other companion animals (dogs or cats) microchipped?

[Single response]

- Yes
- No
- Do not know
- Some

- Other

22. If all or some of your other companion animals are not microchipped, state why.
[Open response]

____

23. Was the animal vaccinated against rabies within the past 3 years?
[Single response]

- Yes
- No
- Do not know
- Performed during the study
- Other

24. If the animal was not vaccinated against rabies, state why.
[Open response]

____

25. Do you regularly use antiparasitic products?
[Multiple responses permitted]

- We do not use them
- Yes, against worms (endoparasites)
- Yes, against ectoparasites (fleas and ticks)
- Other

26. If you do not use antiparasitic products, state why.
[Open response]

____

27. Is the animal neutered?
[Single response]

- Yes
- No
- Do not know
- Performed during the study
- Other

28. If the animal is not neutered, state why.
[Open response]

____

29. Is the animal used for breeding?
[Multiple responses permitted]

- Yes, there is a registered breeding establishment (kennel/cattery)
- Yes, but the breeding establishment is not registered
- The animal(s) are not used for breeding
- Accidental mating sometimes occurs
- There have been unplanned litters (females)
- Other

30. How many litters has the animal had? Females only.

[Open response]

____

______

31. What happens to the offspring?

[Multiple responses permitted]

- Kept by the owner
- Given to relatives or acquaintances
- Given away through advertisements
- Sold
- Surrendered to an animal shelter
- Other

E. Animal Nutrition and Living Environment

32. What is the animal fed?

[Multiple responses permitted]

- Commercial pet food from a grocery shop (e.g., Pedigree, Whiskas, etc.)
- Pet food from a pet shop (premium or super-premium class)
- Raw meat
- Bones
- Porridge
- Human food scraps
- Other

33. Assess how adequately the animal's nutritional needs are met.

[Single response]

- The animal consistently receives sufficient food and water
- Food and water are consistently slightly insufficient
- The animal receives >20% less food and water than required (e.g., a reduced portion and never fully quenches its thirst)
- The animal receives >30% less food and water than required (e.g., a portion reduced by one third and does not drink enough)
- The animal receives >50% less food and water than required (e.g., half a portion, or is fed and watered only every other day)
- The animal receives almost no food or water (>80% less than required)
- The animal frequently goes hungry and is not provided water
- The animal constantly goes hungry and is not provided water
- Other

34. Assessor question: how adequately are the animal's nutritional needs met?

[Single response]

- Not anorexic or dehydrated
- Anorexic, with minimally reduced skin turgor
- Anorexic, with moderately reduced skin turgor and slightly dry mucous membranes

- Anorexic, with markedly reduced skin turgor and dry mucous membranes
- Anorexic, with severely reduced skin turgor and very dry mucous membranes

35. Where is the animal kept?

[Multiple responses permitted]

- Indoors
- Outdoors, in an enclosure
- Outdoors, chained or tethered
- Outdoors, roaming freely
- Other

36. Owner question: on a scale from 1 to 10, assess the animal's living environment.

[Single response; scale from 1 to 10]

Scale options: 1 2 3 4 5 6 7 8 9 10

1 – The animal has good control over its environment, can make a range of choices, and the environment is highly predictable.

2 – The animal has good control over its environment and can make a range of choices, but the environment is less predictable.

3 – The animal has control over its environment, can make some choices, and the environment is moderately predictable.

4 – The animal has some control over its environment, can make some choices, and the environment is partly predictable.

5 – The animal has limited control over its environment, can make few choices, and the environment has little predictability.

6 – The animal spends several hours per day in an unpredictable environment and can make some choices.

7 – The animal spends most of the day in an unpredictable environment and can make very few choices.

8 – The animal spends almost all of its time in an unpredictable environment and can make very few choices.

9 – The animal lives in a highly unpredictable environment and can make very few choices.

10 – The animal lives in an extremely unpredictable environment and cannot make any choices.

37. Assessor question, if the living environment can be evaluated on site: on a scale from 1 to 10, assess the animal's living environment.

[Single response; scale from 1 to 10]

Scale options: 1 2 3 4 5 6 7 8 9 10

1 – The animal has good control over its environment, can make a range of choices, and the environment is highly predictable.

2 – The animal has good control over its environment and can make a range of choices, but the environment is less predictable.

3 – The animal has control over its environment, can make some choices, and the environment is moderately predictable.

4 – The animal has some control over its environment, can make some choices, and the environment is partly predictable.

5 – The animal has limited control over its environment, can make few choices, and the environment has little predictability.

6 – The animal spends several hours per day in an unpredictable environment and can make some choices.
7 – The animal spends most of the day in an unpredictable environment and can make very few choices.
8 – The animal spends almost all of its time in an unpredictable environment and can make very few choices.
9 – The animal lives in a highly unpredictable environment and can make very few choices.
10 – The animal lives in an extremely unpredictable environment and cannot make any choices.

38. The animal has interesting, enriching activities (physical activity, stimulating toys, etc.).
[Single response]

- 2 hours per day or more
- 1–2 hours per day
- Up to 1 hour per day
- Up to 30 minutes per day
- Up to 15 minutes per day
- Several times per week
- Once per week
- Monthly
- Rarely has such activity
- Has no such activity

39. Owner question: on a scale from 1 to 10, assess how well the animal's social needs are met.
[Single response; scale from 1 to 10]
Scale options: 1 2 3 4 5 6 7 8 9 10

1 – Has very high-quality social interactions with people and other animals every day.
2 – Has very high-quality social interactions with people and other animals on most days.
3 – Has good-quality social interactions with people and other animals every day.
4 – Has good-quality social interactions with people and other animals on most days.
5 – Has good-quality social interactions with people and other animals every week.
6 – Has moderate-quality social interactions with people and other animals every week.
7 – Is isolated on most days, but sometimes has moderate-quality social interactions with people and other animals.
8 – Is isolated on most days, but sometimes has poor-quality social interactions with people and other animals.
9 – Is isolated for 50% of the day and has poor-quality social interactions with people and other animals during the remaining time.
10 – Is socially isolated at all times.

40. Owner question: on a scale from 1 to 10, assess the stress the animal usually experiences.
[Single response; scale from 1 to 10]
Scale options: 1 2 3 4 5 6 7 8 9 10

1 – Rarely encounters stressors.

2 – Encounters stressors a couple of times per year.
3 – Encounters stressors every few months.
4 – Encounters stressors monthly.
5 – Encounters stressors weekly.
6 – Encounters stressors several times per week.
7 – Encounters stressors daily.
8 – Encounters stressors for more than 50% of the day.
9 – Encounters stressors for more than 75% of the day.
10 – Experiences stress constantly.

41. Overall, how do you rate the animal's living environment?
[Single response; 1 = very poor and 10 = very good]
Scale options: 1 2 3 4 5 6 7 8 9 10

42. Overall, how do you rate the animal's health?
[Single response; 1 = very poor and 10 = very good]
Scale options: 1 2 3 4 5 6 7 8 9 10

43. Overall, how do you rate the animal's welfare?
[Single response; 1 = very poor and 10 = very good]
Scale options: 1 2 3 4 5 6 7 8 9 10

44. Assessor's additional notes on the animal's living environment and nutrition.
[Open response]

____

______
