# Supplementary material for: Responsible Ownership and Welfare Indicators for Dogs and Cats in Rural Lithuania: Self-Reported Practices and In-Person Assessment
Source: Animals (Basel). 2026 Sep 23;16(19):2988. doi: 10.3390/ani16192988 (PMC13663609; doi:10.3390/ani16192988)
Supplement: Supplementary file 1 [file animals-16-02988-s001.zip › Supplementary Material S3.pdf]

Supplementary Material S3

Adapted Animal Welfare Assessment Form Based on the Animal Welfare Assessment Grid (AWAG)

Animal ID: ______________________________

Physical

Mobility

Assessor-rated

Score	Descriptor
1	the animal has very good mobility with no lameness or stiffness and is normally active or has normal energy
2	very good mobility with occasional mild stiffness and is normally active
3	good mobility with short bouts of stiffness
4	good mobility with generalized stiffness
5	moderate mobility, stiffness but frequently active
6	moderate mobility, stiffness and less active
7	poor mobility, stiffness and less active
8	very poor mobility, stiffness and minimally active
9	very poor mobility, stiffness and not at all active
10	Non-ambulatory and cannot move without assistance or support

Body condition

Assessor-rated

Score	Descriptor
1	ribs easily palpable without pressure, with minimal fat covering, waist easily noted and evident abdominal tuck
2	ribs fairly easy to palpate without pressure with thin fat covering and evident abdominal tuck from above
3	slight fat covering, slight pressure needed to palpate ribs, waist observable from above
4	slight covering of fat, slight waist observable from above, can palpate ribs with pressure needed
5	moderate covering of fat, waist discerned from above but not prominent, can palpate ribs with pressure
6	excess covering of fat, no discernible waistline and difficulty palpating ribs
7	(overweight) heavy fat present and slight abdominal distension, difficult to palpate ribs or (underweight) ribs and shoulder visible with little fat
8	(overweight) heavy fat present with abdominal distension, cannot palpate ribs or (underweight) ribs, lumbar vertebrae and pelvic bones somewhat visible with little detectable fat
9	(overweight) very heavy fat present with obvious abdominal distension, cannot palpate ribs or (underweight) ribs, lumbar vertebrae and pelvic bones easily visible with very little fat
10	Massive fat deposits over neck thorax, spine, limbs and base of tail with obvious abdominal distention, cannot palpate ribs or ribs, lumbar vertebrae, pelvic bones and all bony prominences evident from a distance. No discernible body fat and obvious loss of muscle mass

Clinical assessment

Assessor-rated

Score	Descriptor
1	clinically healthy, no injury or sign of disease

Score	Descriptor
2	mild transient subclinical symptoms or injury but has no evident behavior change or impact on welfare
3	mild transient clinical symptoms or injury with mild transient behavior change and impact on welfare
4	mild clinical symptoms or injury with mild behavior change and impact on welfare
5	moderate transient clinical symptoms or injury with some behavior change and impact on welfare
6	moderate clinical symptoms or injury with moderate behavior change and impact on welfare
7	moderate/severe disease or injury with moderate behavior change and impact on welfare
8	moderate/severe disease or injury with severe behavior change and impact on welfare
9	severe disease and clinical symptoms or injury with severe of behavior change and impact on welfare
10	Extreme disease and clinical symptoms or injury with extreme behavior change and impact on welfare

Eating and drinking

Owner-reported; assessor observations were recorded where available

Score	Descriptor
1	eating and drinking as normal
2	food and/or water consumption is minimally reduced
3	mild to moderate reduced food/water (>20%)
4	moderately reduced food/water (>30%)
5	moderately reduced food/water (>50%)
6	severely reduced food/water (>80%)
7	anorexic, has minimal loss of skin turgor
8	anorexic, has moderate loss of skin turgor, somewhat dry mucous membranes
9	anorexic, has considerable loss of skin turgor, dry mucous membranes OR severe hunger/thirst
10	Anorexic, has major loss of skin turgor, extremely dry mucous membranes OR severe and constant hunger/thirst

Psychological

Aggression toward caregiver

Assessor-rated; cat-inclusive behavioural terms were added

Score	Descriptor
1	none
2	occasionally growls or hisses, is predictable and trigger avoided
3	occasionally growls or hisses, is predictable but trigger not always avoided
4	occasionally growls or hisses, is predictable but trigger rarely avoided
5	occasionally snaps, bites, or scratches, is predictable and trigger avoided
6	occasionally snaps, bites, or scratches, is predictable but trigger not always avoided
7	occasionally snaps, bites, or scratches, is predictable but trigger rarely avoided
8	bites or scratches, is somewhat predictable and trigger largely avoided
9	bites or scratches, is somewhat predictable and trigger not avoided
10	severe bites or scratches that are unpredictable

Aggression toward unfamiliar people

Assessor-rated; cat-inclusive behavioural terms were added

Score	Descriptor
1	none
2	occasionally growls or hisses, is predictable and trigger avoided
3	occasionally growls or hisses, is predictable but trigger not always avoided
4	occasionally growls or hisses, is predictable but trigger rarely avoided
5	occasionally snaps, bites, or scratches, is predictable and trigger avoided
6	occasionally snaps, bites, or scratches, is predictable but trigger not always avoided
7	occasionally snaps, bites, or scratches, is predictable but trigger rarely avoided
8	bites or scratches, is somewhat predictable and trigger largely avoided
9	bites or scratches, is somewhat predictable and trigger not avoided
10	severe bites or scratches that are unpredictable

Fears and anxieties frequency

Owner-reported

Score	Descriptor
1	rarely encounters stressors
2	encounters stressors a couple of times a year
3	encounters stressors multiple times a year
4	encounters stressors monthly
5	encounters stressors weekly
6	encounters stressors several times weekly
7	encounters stressors daily
8	encounters stressors over 50% of the day
9	encounters stressors over 75% of the day
10	encounters constant stressors

Reaction to stressors

Assessor-rated

Score	Descriptor
1	displays minimal signs of fear and anxiety when encounters potential stressors
2	shows signs of fear to stressors and returns to normal <30 s
3	shows signs of fear to stressors and returns to normal in minutes
4	shows signs of fear to stressors and some minor and returns to normal after 10 minutes
5	shows signs of fear to stressors and returns to normal after 30 min
6	shows signs of fear to stressors and takes up to an hour to return to normal
7	shows signs of fear to stressors and takes several hours to return to normal
8	shows signs of fear to stressors and takes most of the day to return to normal
9	shows signs of fear to stressors and takes several days to return to normal
10	shows signs of fear to stressors and is always anxious

Environment

Choice, control, and predictability

Owner-reported and, where the assessment occurred in the animal's home, assessor-rated

Score	Descriptor
1	has good control over their environment and can make a range of choices, has highly predictable environment
2	has good control over their environment and can make a range of choices, mostly has predictable environment
3	has some control over environment, can make some choices, has mostly predictable environment
4	has some control over environment, can make some choices, has some predictability
5	has little control over environment, can make some choices, has little predictability
6	spends several hours in an unpredictable environment, can make some choices
7	spends time in an unpredictable environment, can make few choices
8	spends time in an unpredictable environment, can make few choices
9	spends time in an unpredictable environment, can make very few choices
10	spends almost all of their time in highly unpredictable environment, cannot make any choices

Enrichment

Owner-reported

Score	Descriptor
1	engaged in multiple forms of enrichment for over 2 h daily
2	engaged in multiple forms of enrichment for 1-2 h daily
3	engaged in multiple forms of enrichment for up to 1 h daily
4	engaged in enrichment for up to 30 mins daily
5	engaged with enrichment for <15 mins daily
6	somewhat engaged with enrichment several times weekly
7	somewhat engaged with enrichment weekly
8	poorly engaged with enrichment monthly
9	rarely engages with any forms of enrichment
10	has no enrichment or does not engage with enrichment

Social

Owner-reported; species-neutral wording was used

Score	Descriptor
1	has high-quality social interactions daily
2	has high-quality social interactions most days
3	has good-quality social interactions daily
4	has good-quality interactions most days
5	has good-quality interactions weekly
6	has moderate-quality interactions weekly
7	the animal is socially isolated most days and has moderate-quality interactions in between
8	the animal is socially isolated most days and has poor social interactions in between
9	the animal is socially isolated for 50% of each day and has poor social interactions the rest of the time
10	the animal is constantly socially isolated

Procedural

Behavior during assessment

Assessor-rated

Score	Descriptor
1	is calm and actively seeks interaction from assessor/s
2	is mostly relaxed and shows mild signs of stress to few triggering events
3	is somewhat relaxed and shows mild signs of stress to some triggering events
4	is not relaxed and shows moderate signs of stress to few triggering events
5	shows moderate signs of stress to some triggering events
6	shows moderate signs of stress to all triggering events
7	shows major signs of stress to few triggering events
8	shows major signs of stress to some triggering events
9	shows major signs of stress to all triggering events
10	cannot cope being in the environment, is extremely shut-down or aggressive and shows major signs of stress

Handling during assessment

Assessor-rated when handling or a procedure occurred; cat-inclusive behavioural terms were added

Study-specific option: No procedure performed (coded 0 in the analytical dataset).

Score	Descriptor
1	displays minimal signs of stress when handled, is calm and tolerates being handled well
2	minimal movement when handled, sometimes licks lips, yawns or shows other appeasement behavior
3	minimal movement when handled, licks lips, yawns, or shows appeasement behavior frequently
4	some slow movement when handled, turns head away from handler, slow panting, displays more than two signs of stress such as ears back and tail down
5	moderate movement when handled, fast panting, displays more than two signs of stress such as ears back, tail tucked and furrowed brow
6	some attempt to escape, fast movements, tense body and tense closed mouth
7	moderate attempts to escape, fast movements or frozen and staring, tense and trembling
8	strong attempts to escape when handled or frozen, lifts lips and shows teeth
9	will violently attempt to escape when handled or frozen, growls, hisses, barks or vocalises
10	cannot be handled, growls or hisses and attempts to bite or scratch when approached
